# Embracing complexity and uncertainty to create impact: exploring the processes and transformative potential of co-produced research through development of a social impact model

**DOI:** 10.1186/s12961-018-0375-0

**Published:** 2018-12-11

**Authors:** Kate Beckett, Michelle Farr, Anita Kothari, Lesley Wye, Andrée le May

**Affiliations:** 1 The University of The West of England, Centre for Child & Adolescent Health, Oakfield House, Oakfield Grove, Bristol, BS8 2BN United Kingdom; 2 NIHR CLAHRC West, University Hospitals Bristol NHS Foundation Trust, 9th Floor, Whitefriars, Lewins Mead, Bristol, BS1 2NT United Kingdom; 3 Population Health Sciences, Bristol Medical School, University of Bristol, Canynge Hall, 39 Whatley Road, Bristol, BS8 2PS United Kingdom; 4 School of Health Studies, University of Western Ontario, Health Sciences Building Room 222, London, ON N6A 5B9 Canada; 5 Centre for Academic Primary Care, Population Health Sciences, Bristol Medical School, University of Bristol, Canynge Hall, 39 Whatley Road, Bristol, BS8 2PS United Kingdom; 6 Faculty of Health Sciences, University of Southampton, University Road, Southampton, SO17 1BJ United Kingdom

**Keywords:** Knowledge mobilisation, co-production, integrated knowledge translation, knowledge translation, impact framework, case studies

## Abstract

The potential use, influence and impact of health research is seldom fully realised. This stubborn problem has caused burgeoning global interest in research aiming to address the implementation ‘gap’ and factors inhibiting the uptake of scientific evidence. Scholars and practitioners have questioned the nature of evidence used and required for healthcare, highlighting the complex ways in which knowledge is formed, shared and modified in practice and policy. This has led to rapid expansion, expertise and innovation in the field of knowledge mobilisation and funding for experimentation into the effectiveness of different knowledge mobilisation models. One approach gaining prominence involves stakeholders (e.g. researchers, practitioners, service users, policy-makers, managers and carers) in the co-production, and application, of knowledge for practice, policy and research (frequently termed integrated knowledge translation in Canada). Its popularity stems largely from its potential to address dilemmas inherent in the implementation of knowledge generated using more reductionist methods. However, despite increasing recognition, demands for co-produced research to illustrate its worth are becoming pressing while the means to do so remain challenging. This is due not only to the diversity of approaches to co-production and their application, but also to the ways through which different stakeholders conceptualise, measure, reward and use research. While research co-production can lead to demonstrable benefits such as policy or practice change, it may also have more diffuse and subtle impact on relationships, knowledge sharing, and in engendering culture shifts and research capacity-building. These relatively intangible outcomes are harder to measure and require new emphases and tools. This opinion paper uses six Canadian and United Kingdom case studies to explore the principles and practice of co-production and illustrate how it can influence interactions between research, policy and practice, and benefit diverse stakeholders. In doing so, we identify a continuum of co-production processes. We propose and illustrate the use of a new ‘social model of impact’ and framework to capture multi-layered and potentially transformative impacts of co-produced research. We make recommendations for future directions in research co-production and impact measurement.

**Electronic supplementary material:**

The online version of this article (10.1186/s12961-018-0375-0) contains supplementary material, which is available to authorized users.

## Introduction

Globally, factors inhibiting the uptake of scientific evidence and, hence, the ability of health research to influence healthcare policy and improve practice are increasingly acknowledged [1–3]. Consequently, recognition of research co-production as a means to generate, and apply, rich implementable knowledge for healthcare policy and practice is expanding rapidly. Nevertheless, its impact remains unclear [4], partly due to the range of approaches under the co-production banner and current emphases within impact measurement. This paper is informed by published and grey literature, analysis of the authors’ co-produced research and insights that draw on our collective research experiences in Canada and the United Kingdom, generated through six iterative author workshops. It explores (1) the emergence and processes of research co-production, (2) how the co-production of research can increase research impact, (3) the mechanisms involved and (4) how this impact can be captured. We explicitly chose this approach to give us an opportunity to re-experience and collectively explore the benefits and challenges of co-production. It enabled us to identify a continuum of co-production processes and investigate their various impacts using a new analytic framework and case studies from our research. In this paper, we consider the paradigmatic implications of co-production methods and their potential for securing wider, more sustainable returns on investments in research. We propose a ‘social model of impact’ as an adjunct to existing more economic measures. We conclude by making recommendations for future directions in research co-production and for optimising and capturing the impact of co-produced research.

## Background

Few contest that research has the potential to improve the quality, effectiveness and consistency of healthcare. However, despite vast amounts of energy and funds directed globally towards improving the research evidence base behind policy and practice, there are clear limitations to existing methods of knowledge generation, dissemination and uptake, and thus our ability to improve healthcare quality by means of research [1–3]. Indeed, in the United Kingdom alone, despite an annual expenditure on health research of approximately £8 billion [5], most research fails to have a significant or lasting effect on policy or practice. Within a global climate of increased demand and finite resources, this return on investment, both financial and intellectual, is unacceptably poor. This has led to considerable effort from numerous stakeholders, resulting in a proliferation of approaches to transform research evidence into implementable practices.

Over time, these dilemmas have resulted in changes to the way in which the ‘gap’ between research and practice (or policy), and the best means to span it, have been conceptualised and addressed [6]. Earlier assumptions were that the passage of research evidence into practice was largely linear and rational, and all that was required was to teach practitioners how to critically assess research and build organisational support (i.e. sufficient push or pull) [1]. This has been replaced with more complex, social and relational models that seek to address the messy contextual realities of real-world healthcare [7–10]. Simultaneously, debate has highlighted yawning gaps between academic and health service cultures, timelines, interests and rewards, and the resulting need for collaborative methods, linkage and bridging skills [11, 12]. Questions have emerged about the nature, and ownership, of knowledge required for effective healthcare, and the processes by which it is generated and modified [2, 13, 14]. Knowledge, it is clear, is not an objective immutable product that can be packaged and transferred between contexts, but is dynamic, changeable, contested and politically imbued [15].

Recognition of the need for a richer, more inclusive ‘evidence’ base for real-world healthcare (including service user and practitioner perspectives and stories), which engages with and better reflects the emotional, relational, organisational, practical and rational aspects of care and policy [16], is not new [17]. However, the drivers for such a change have gained momentum in recent years. For example, ethnographic research shows that clinical decisions are informed by ‘clinical mindlines’ containing evidence from multiple sources (including tacit and experiential knowledge and research) [14, 18]. Mindlines are learned, modified and applied using social means within, for example, practitioner ‘communities of practice’ [14]. They are tested in practice and equip practitioners with the necessary ‘contextual adroitness’ for clinical decision-making and to address healthcare’s multiple realities and demands [14]. Recent extensions to this work show how different agents/agencies engaged in the creation, policy-setting, use, or outcomes of health research have their own individual and collective mindlines relating to their specific world [13, 19]. The challenge for research in improving the quality of healthcare is therefore to acknowledge and utilise, rather than attempt to control this complexity [15], and to create social contexts and research approaches in which knowledge, practice and policy can be interrogated, modified and learned. Knowledge mobilisation (KM) is evolving to meet these challenges, but its evolution and expression have taken different forms, as demonstrated below.

### Knowledge mobilisation (KM): definition and approaches

KM (sometimes called knowledge translation) is an umbrella term, defined broadly as the sharing of knowledge. Advances in KM over the past 20 years have led to new ways of thinking, driving new research methods and organisational structures to promote knowledge sharing – each with its own, underpinning rationale and purported mechanism(s) of action [3]. Consequently, Davis et al. [3] systematically mapped diverse KM strategies and structures employed in the English NHS and its international comparators against six conceptual domains, namely (1) purpose(s) and goals (implicit or explicit), (2) knowledge types used, (3) connections and configurations, (4) people, roles and positions, (5) actions and resources available, and (6) context of operation. This led to the identification of eight KM archetypes, described from A to H, which provide a useful platform for agencies or researchers to compare and inform their KM activities [3].^.^ Archetypes A, F and G represent strategies at opposite ends of Davies et al.’s [3] conceptual map (Box 1).

Davies et al. [3] do not suggest these archetypes are mutually exclusive, which strategies are most likely to be effective or claim superiority of any one approach. However, activities that broadly conform to Archetypes F and/or G combine elements that appear to directly address many problems facing the uptake of evidence. These approaches also offer means to embrace the complexity and diversity of researcher and stakeholder mindlines and help in developing the ‘contextual adroitness’ required for real world policy and practice. In the remainder of this paper, we are therefore interested in KM activities that explicitly emphasise research co-production (or integrated knowledge translation (IKT)), network building, broad inclusive knowledge sources and context, i.e. those that broadly conform to Davies et al.’s [3] Archetypes F and/or G. We start with a discussion of the principles and practices of research co-production and IKT.

### Principles and practices of research co-production

Co-production can be defined as “*a process through which inputs from individuals who are not* [generally] *‘in’ the same organisation are transformed into goods and services*” ([20], p. 1073). In co-production, both ‘producers’ and ‘users’ aim to collaborate equitably in the co-production process [21]. Knowledge users are active agents not passive recipients, and their knowledge is valued equally [22]. Co-production literature frequently focusses on the co-production of services by policy-makers/practitioners and the public/service users. However, it is increasingly applied to the co-production of knowledge by researchers, policy-makers, managers, practitioners, and/or service users and their carers/families. The co-production of research is a type of KM in which a “*plurality of knowledge sources are combined, usually to address specific problems*” ([23], p. 221); together, they may achieve more than they can alone [22]. Research co-production ideally adheres to the following key principles: sharing of power, including all perspectives and skills, valuing the knowledge of everyone, reciprocity and building relationships [24]. Outputs of co-produced research can be transformed by knowledge-user participation; consequently, they may better meet users’ needs and support decision-making and implementation in the local setting [22]. Research co-production starts from a different epistemological and ontological stance to traditional or reductionist approaches to knowledge generation and dissemination; to illustrate, Table 1 contrasts these approaches using Davies et al.’s [3] six domains.

**Table 1:** Using Davies et al. [3] conceptual domains to compare research co-production with more reductionist approaches

Conceptual domain	Co-production	Reductionist approaches
Knowledge types	Broad, inclusive, range of types. Includes research knowledge produced within local contexts that may be applied more widely after review. Values and emphasises explicit, actionable, tacit and experiential knowledge	Research knowledge produced independently of those working in the situation being researched; implies a ‘hierarchy of evidence’
Actions and resources	All mechanisms in use, especially interaction, social influence, facilitation, dissemination, training and education. Embraces complexity, uncertainty and dissonance. Multiple approaches to dissemination	Randomised controlled trials predominate as ‘gold standard’. End of project dissemination mainly via guidelines and peer-reviewed articles are the norm
Purpose and goals	Knowledge-driven, problem-solving, interactive use. Aims at shaping a wide range of outcomes, fosters unexpected types and sources of impact. Capacity-building and shared learning. Emphasis on research and implementation	To generate generalisable facts using rigorous (and ideally controlled) methods largely to answer specific pre-determined questions or test hypotheses. Means to mobilise or implement results not always emphasised nor made explicit
Connections and configurations	Relationship models; systems models	Linear models (may include push and pull)
People and roles	Different stakeholders centrally involved on an equal basis, including researchers, practitioners, managers, policy-makers, service users and the public	Distinction between researchers as ‘knowledge producers’ and policy-makers, managers, practitioners or service users as ‘knowledge users’ or ‘recipients’. Researchers as experts
Context	Emphasis on internal and external context as active ingredients to change. Responsive to dynamic circumstances	Attempts to exclude contextual factors by controlling for them where possible, i.e. they remain in the background

However, research co-production is a complex social and political process [25] and not, as sometimes described, a simple panacea for the poor uptake of research evidence. The following section explores key elements or mechanisms and known challenges of research co-production.

### Key elements or mechanisms, and challenges in research co-production

To begin a process of research co-production, problems need to be collaboratively identified. Key contributors to the co-production process need personal qualities, such as openness, tolerance and flexibility [23], and commitment to collaboration, communication, rapport building and negotiation [26]. Co-production of knowledge requires time, resources, blurring of boundaries and methodological exploration [27]. Knowledge brokers might also be implicated as key actors in collaborative processes as they can overcome barriers related to relationship development and staff turnover. There is evidence that knowledge brokers currently do enact mechanisms (e.g. meetings, dialogues, relationship-building) to support collaborations [28].

Challenges for co-production include conflicting values, institutional rigidity and risk aversion, ensuring accountability, and shortage of capacity and incentives [2]. Valuing different forms of knowledge is vital [23, 27], alongside sharing power [29] and working towards an ideal of equal relations [22, 25]. This can be demanding, as power and politics need careful negotiation and navigation [23] and different stakeholders and groups have their own cultural values and language, which can reinforce hierarchies [27]. Traditional power-holders may need to relinquish influence [30] and unequal power relations need to be identified and addressed to avoid reproducing gender, racial/ethnic and socioeconomic inequalities [31]. For example, the power and privilege conferred on researchers by their university affiliations may potentially affect collaborative processes with other stakeholders and communities [29, 31]. Representatives of power-holding institutions need to take responsibility to work towards equitable partnership with patients, communities and the public [29].

In order to realise tangible impacts from co-produced research, collaborative processes should involve different stakeholders rather than only those with greater power [27]. However, evidence also suggests that involving those who have the authority to implement change within organisational and policy systems is key, as they have specific expertise in the area, and understand the likely facilitators and barriers to implementation [32]. Attempts at collective action in implementation might be determined by the deliberate alignment of several features, including foundational relationships, vision, values, structures and processes, and views about the nature of the collaboration and implementation [30].

### Maintaining rigour in co-produced research

As discussed, research co-production is neither a simple nor unidimensional process. If one considers the key elements and challenges (above) of co-production, the inherent difficulties in achieving rigour and robustness in design, and thereby outcomes, are clear to see. Thus, assessing both rigour, relevance and flexibility at the proposal stage are critical if value for money as well as likely impact are to be obtained. In a move towards distinguishing between high quality and poorly conceived co-production research, the United Kingdom N8 partnership recently proposed an 11-area evaluative framework to enable funders (and others) to evaluate this type of research proposal [25]. These criteria include the need to focus on partnerships rather than projects, have experience and understanding of participatory engagement and facilitation, see evidence of reflective learning, and understand how opportunities for translation to support effective change are to be enacted [25].

Research co-production therefore goes far beyond consultation. Its growing popularity and recognition reflect its ability to achieve both rigorous and relevant findings [25]. It is also important to note that, while the term ‘research co-production’ is increasingly used, collaborative research is rooted within diverse traditions and rationales, including participatory, collaborative and community engaged research, participatory/action research, communities of practice, civil rights, feminist and disability rights, and open innovation approaches [33]. Furthermore, there are global variations in its manifestation and in the terms used, for example, IKT [32] in Canada (see below).

### Integrated knowledge translation (IKT)

IKT is an increasingly prominent form of co-production in Canada, which actively tackles the need for early KM and translation [34, 35]. IKT is defined as an approach to collaborative research, in which researchers work with knowledge users who identify a problem and have the influence, and sometimes authority, to implement the knowledge generated through research [32]. Knowledge users “*function as active partners to generate research from conceptualisation to implementation, rather than be passive recipients of research or research products*” [34]. Knowledge users go beyond influencing the stages of research – they are co-investigators who carry out the research process in partnership with researchers, starting with the selection of a research question [36, 37]. Both researchers and knowledge users bring their expertise (methodological, contextual, topic related) to the project to generate research findings. In emphasising the role of knowledge-users specifically selected for their “*authority to invoke practice or policy change*” [33, 34], IKT brings issues of power to the fore. However, recent scoping reviews of IKT strategies reveal that, alongside other forms of research co-production, the area is theoretically undeveloped, requires greater attention to processes of engagement, and needs to establish stronger evidence between IKT models and outcomes [34, 38].

### A continuum of research co-production

Co-produced research allows research ‘users’ to influence the production, mobilisation and transformation of knowledge at different stages within the research process, e.g. during the development of research questions, methods, data collection and analysis, which may help to then influence its application, outputs and outcomes, as opposed to being passive end-point recipients. Ideally, co-production occurs at all stages of the knowledge generation and application process and with all stakeholders, but this may be difficult to achieve and is the subject of much debate. However, in their recent review of IKT studies, Gagilardi et al. [34] found that the involvement of stakeholders tends to be under-described, making it difficult to conclude whether ideal, full involvement leads to better outcomes compared to selective involvement at particular stages.

**Fig. 1:**
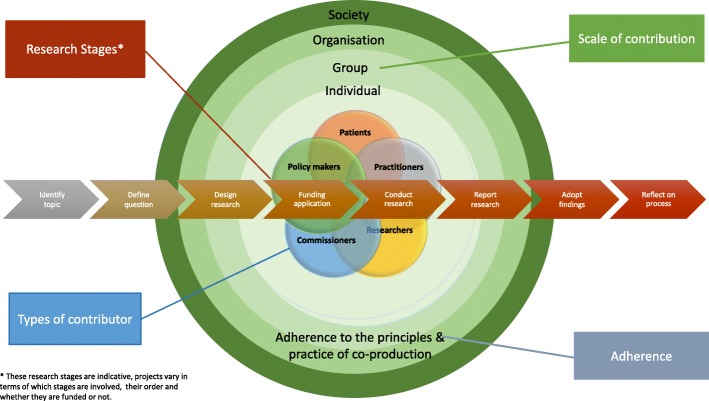
The research co-production continuum. This diagram shows that the degree to which research can be said to be ‘co-produced’ is a factor of how many research stages are co-produced, the types of stakeholder involved, the scale of their contribution, and ‘adherence’ to the principles and practice of co-production. *For example: a university designed and conducted research project in which co-production between individual researchers and practitioners occurs at the ‘define question’ stage only; power imbalances persist at one end of the continuum whereas at the other there is major contribution from all stakeholders in the co-production of all research stages, adhering to the principles and practices of co-production*

Our experience suggests that co-produced research is situated along a continuum in terms of the number of research stages, the way stakeholders are involved in co-production, the project scope and scale, and the degree of adherence to the principles and practice of co-production achieved (Fig. 1).

Note that, in developing the dimensions of this continuum, we chose ‘adherence to the principles of co-production’ after much deliberation as a means to capture the ‘authenticity’ of co-production and extent to which it incorporated the key principles of co-produced research [24]. We suggest that reflecting on and evaluating the extent to which a research project has been co-produced may also be supported by using the N8 partnership evaluative framework [25]. Models that are designed to evaluate public involvement in research may also be helpful to reflect on the extent to which people are involved and influential in co-produced research [39]. Further work is needed to develop criteria to determine the extent of co-production within research and how we evaluate and assess co-produced research [25].

In this paper, we focus on researchers working with policy-makers, organisations, practitioners and/or service users or their carers to co-produce research knowledge at any point in the research process (i.e. at any point on our continuum). The remaining sections focus on capturing the impact of this type of research.

### Issues in measuring the impact of co-produced research

To demonstrate impact, we need to understand the various terms used to describe impact (Table 2) and to be able to capture how and where it occurs. However, determining research impact is difficult and complicated by the demands of different target audiences for evidence of different sorts of impact. Consequently, research-to-impact measurement has mushroomed, resulting in “*a confusing array of models that draw on different epistemological assumptions about the link between research and impact*” ([6], p. xxii). Research co-production approaches are likely to be more aligned philosophically with impact models that are critical and participatory and embrace a range of impacts, such as capacity development or network building, in addition to traditional impacts focused on behaviour change or economic benefit. They need to emphasise the “*non-linearity, messiness, and unpredictability of the collaborative knowledge production process*” ([6], p. 59). Currently, effective means to systematically evaluate and capture these more multifaceted impacts remain unclear.

**Table 2:** Definition of impact and associated terms, with examples from Case Study 1 (CS1: Additional file 1)

Term	Definition	Example
Outputs	Products, such as journal articles, conference presentations, guidelines, recommendations, summaries and tools	• Andrews N, Gabbay J, le May A, Miller E, O’Neill M, Petch A. Developing evidence-enriched practice in health and social care with older people. 2015. Joseph Rowntree Foundation, York (also see ‘Box 3 & 4 Case study [57, 58] at the end) • CS1 will also inform an impact case study in the next United Kingdom Research Excellence Framework Assessment (http://www.hefce.ac.uk/rsrch/REFimpact/)
Uses	Instrumental, conceptual or symbolic use of the outputs	Practice changes across all sites and multiple alterations to delivery/content of staff education and development, e.g. related to risk, relationships, working with residents to be more person centred
Outcomes	Identifying what changed as a result of the use of the outputs	Project approach woven into the National Dementia Learning and Development Framework for Wales and informed policy change (Good Work - A Dementia Learning and Development Framework for Wales, Care Council for Wales, Cardiff, 2016; https://socialcare.wales/resources/good-work-dementia-learning-and-development-framework)
Impacts	A collective term encompassing output, uses and outcomes	Participants across all sites reported enhanced wellbeing due to their involvement, indicating development of an ‘enriched environment’ of learning [57]. Participants felt a sense of security, continuity, belonging, purpose, achievement and significance – that they mattered – and that things could change for the better. The evaluation revealed improved relationships, greater networking opportunities, information exchange and increased trust among professionals and between policy-makers, managers, professionals, older people, carers and different sites

The emphasis on measurable, economic and quantifiable impacts and relative neglect of ‘productive interactions’ or social impacts that occur in complex health research systems results in a partial view of the contributory processes and potential impacts of co-produced research. This may reinforce the appeal, to funders and research institutions, of apparently more tangible direct impacts offered by more reductionist models of research. To establish the extent to which co-produced research can affect improvements in health systems and population health, it is imperative that we address the challenges of measuring diverse, positive and negative impacts of this type of research.

To account for these issues, new approaches to studying KM activities, such as co-production and research impact, include ‘complex systems’ approaches incorporating multi-stakeholder networks [7], public value mapping [16] and contribution analysis, which is based on narratives and a wider range of different evidence types [15]. To capture non-linear impacts within co-produced research, we need to understand both processes and outcomes so that we can attribute impacts to the co-produced research [25, 40–42]. For example, as IKT highlights, if we involve decision-makers with authority to make changes, this may facilitate implementation as key stakeholders are already interested and involved. Thus, process and outcome measures to understand co-production within research collaborations are an important development [26]. Other advances include tools such as Barwick’s Knowledge Translation Planning Template [43, 44], which provide a useful framework to measure different types of research impact, including relational. However, research impact is often diffuse, long-term and potentially difficult to track; this becomes more complex within co-produced research. Research impact methods therefore need to account for this complexity and to capture the partnerships and processes involved in the co-production of knowledge between academy, policy-makers, service providers and citizenry [15], public engagement, ‘conceptual impact’ and ‘capacity-building’ [17], and cultural shifts in research and practice institutions [45].

Capturing the breadth of impact in co-produced research clearly requires new emphases and tools. In the following section, we therefore propose, and illustrate the use of, an analytic multi-layered framework with the ability to capture the potential breadth of co-produced research impacts. We offer this as an adjunct to strengthen existing assessments, for example, those already undertaken by the Canadian Institutes for Health Research [46] of health and economic impacts, or sector assessments such as the United Kingdom’s assessment of performance in higher education institutions (the Research Excellence Framework) [47] or assessments by care providers through the adoption of findings into guidelines and policies and their use.

### Towards a research co-production impact framework

In developing a framework for capturing the impact of co-produced research, we were drawn to advances in related fields; for example, in the context of implementation science, complexity and systems approaches highlight multiple levels of influence on implementation, and relationships within and across levels, which lead to different synergies and outcomes [48]. Research implementation can be understood as a series of feedback loops, rather than as a linear process [49]. This means that there may be multiple mechanisms and interactions [50, 51] occurring within an implementation process, taking place at different levels over time, with interdependent relationships between them [48, 52]. Mechanisms of action within research co-production may occur and cause impacts at different levels, these impacts having the potential to become future mechanisms of action, which may initiate further changes over time. Other models explore situational and relational outcomes throughout the life-time of the research [42, 48]. However, most impact frameworks still focus on the end stage of a project after peer-reviewed articles have been published and findings disseminated [41]; these assume changes start at a macro-level filtering through to a meso- and micro-level (i.e. research influences policy, which influences practice). However, the impacts of co-produced research may start at a micro-level involving local policy-makers and practitioners through the research process long before peer-reviewed articles have been published. Indeed, Pawson [52] advocates exploring interactions and events between these different levels over time, and understanding of historical trajectories.

Since co-produced research may have multi-layered nuanced impacts, we have adapted Pawson’s ([52], p. 36–37) notion of context (listed 1–4 below) to inform a preliminary framework for mapping micro to macro levels of impact that can ensue from co-produced research. We have combined this with Pfadenhauer et al.’s [48] conceptualisation of the micro, meso and macro levels to aid understanding.

1. Individual (micro-level) – characteristics of stakeholders, including biological and psychological aspects (i.e. improved mental or physical health, improved practice and skills for practitioners).
2. Groups/networks/interpersonal relations (micro-level) – stakeholder relationships within a system (researcher/practitioner partnerships), practice changes within teams/departments.
3. Organisational or institutional (meso-level) – organisations including rules, norms (culture), capacity-building and organisational structures, funding organisations, educational institutions.
4. Societal or infrastructure (macro-level) - wider social, economic, policy and political impacts. Multiple institutions at a national scale. National public engagement, different elements of social and public value such as justice and equality.

We propose that to understand co-produced research impacts we need to capture and analyse the different elements of 1–4 and how their interactions may create emergent properties. Here, emergence can be described as “*a whole having properties that are more than the sum of its parts*” [50]. To understand and document how impacts are catalysed through co-productive research we need to analyse nonlinear chains of contribution [25] that reflect the dynamism of complex health research systems. We need to consider longer term developments, wider social changes, any unintended consequences and how co-produced research might affect and be affected by different power dynamics.

To develop this preliminary framework, we applied it to six case studies purposefully selected from our own co-produced research. These case studies, from Canada and the United Kingdom, were chosen to ensure maximum variation in terms of their placement on the co-design continuum (i.e. in terms of research stages co-produced, types of contributor, scale of their contribution, and adherence to the principles and practice of co-production). Selection was according to the following method: authors presented several potential co-produced case studies to the group at a face-to-face workshop, we interrogated each one in relation to these key dimensions and collectively chose those for inclusion based on the criteria above. Selection was also guided by an a priori decision to include at least one case study per author and examples from both the United Kingdom and Canada, since we explicitly aimed to generate ideas through past and real-time experience of the challenges and benefits of co-production. Our choices were also clearly limited to the types and scope of projects we as authors had engaged in. Box 2 below summarises the six case studies chosen (full case study summaries, including types of contributor, scale, method and impacts, are included in Additional files 1, 2, 3, 4, 5, and 6 to inform the following analysis and subsequent conclusions and recommendations).

### Applying our impact framework

To analyse these six case studies, we created a grid based on the above framework (Additional file 7) to map (1) contributors and processes involved in our six co-produced research case studies; (2) their impacts (outputs, uses, outcomes); and (3) contributory mechanisms, at each of the four levels (individual, group, organisational, societal). Next, each author analysed their own case study and made notes on the grid; these were subsequently shared, discussed and refined within an extended face-to-face author workshop. This permitted us to combine and synthesise findings from our individual case grids. Finally, these merged findings were analysed to discern broad themes in terms of the relationship between co-produced processes, their impacts and key mechanisms. KB completed the initial phase of this broader impact level analysis, AlM provided secondary independent verification and their combined findings were iteratively questioned and corroborated by other members of the team at subsequent workshops. We found that the impact framework was practical and easy to use; it helped us to simultaneously explore processes, impacts and contributory mechanisms.

#### What we found

While our case studies exemplified different points on the co-production continuum and their impact varied in degree and timing, we found that two distinct impact ‘patterns’ could be distinguished within them all, namely (1) ‘specific level impacts’ and (2) ‘broad impacts’ occurring across all levels. However, as previously observed [50–52], we found the same phenomenon could be both mechanism and impact, e.g. a mechanism may cause an impact, this impact then becomes another mechanism, which causes another impact.

#### Specific level impacts

Specific impacts were found to re-occur in our case studies at some levels, e.g. individual, but not across all levels. Box 3 summarises these impacts.

#### Broad impacts

Broad impacts were found to re-occur across case studies and across levels (individual, group, organisational and societal). Further analysis suggested these broad impacts, occurring at every level, could be categorised according to four overarching themes which we named (1) knowledge required for effective healthcare policy and practice; (2) research for healthcare policy and practice; (3) capacity for research; and (4) nature of impact. Our case studies’ broad impacts are illustrated under these themes in Box 4.

### Paradigmatic impacts arising from co-produced research

The sections above suggest that, to succeed and realise impact research, co-production requires specific skills, time and resources. However, by extrapolating from our case studies within our workshops we also noticed that, where successful, the multi-level processes, impacts and momentum of co-production also combined to promote and sustain much broader change. Indeed, it became apparent that research co-production potentially leads to a fifth level of impact, which is more conceptual and discursive than the original four. We have named this level ‘paradigmatic’ as it has potential to modify ways of understanding the world and shift frames of reference. This may involve wider cultural struggles over what is considered ‘legitimate’ knowledge and challenging the ‘cultural hegemony of powerful groups’ [53], resulting in a culture shift and realignment of our relationship to knowledge, research and healthcare practice and policy. These significant effects are poorly captured with current impact frameworks and highlight the need for a ‘social model of impact’ to complement those already in use. Table 3 illustrates the paradigmatic implications of research co-production emerging from our case studies and deliberations.

**Table 3:** Paradigmatic implications of research co-production

Processes	Impacts
1. Emergence of new ideas, methods and relationships	• Proliferation of new ideas • Knowledge greater than the sum of its parts • Recognition and shift towards new research methods to facilitate co-production/integrated knowledge translation • Greater appreciation of blending techniques within academic institutions • Stronger links and understanding developed between multiple practice and academic disciplines • More diverse, enduring and representative engagement in the processes and outcomes of research, e.g. practitioners and service users being named on or leading further research proposals • Co-design of questions and co-analysis of data aided the transferability and validity of results • Practitioners and patients explicitly recognised for participating in research and contributing to the development of its outputs
2. Transformative synergies as a result of complex sequences of interventions and interactions	• Questions the nature of knowledge • Acknowledges, harnesses and perpetuates the democratisation of knowledge • Challenges the hegemony of reductionist approaches to healthcare research • Enables research that is dynamic, agile and responsive to local contexts and changing circumstances • Embraces complexity, dissonance and uncertainty • Creates rich contextualised evidence from various sources to foster stakeholders’ contextual adroitness and furnish their mindlines with other perspectives • Harnesses the creativity, expertise, experience and energy of people who provide and use services – this can be politically and practically productive • Permits redesign and regulation of services to reflect the needs of people who use and work within them • Places human contextual and emotive issues within research; engages with research users’, generators’ and policy-makers’ emotive and rational selves • Facilitates an ideological shift towards justice and equality rather than hierarchy and power imbalance in the process and outcomes of research • We also discerned the potential for co-production to create a virtuous cycle; a recurring cycle of events, in which learning, innovation and improvement are embedded and continuous, and each cycle increases the benefit of the ones before

### The transformative potential of co-production

**Fig. 2:**
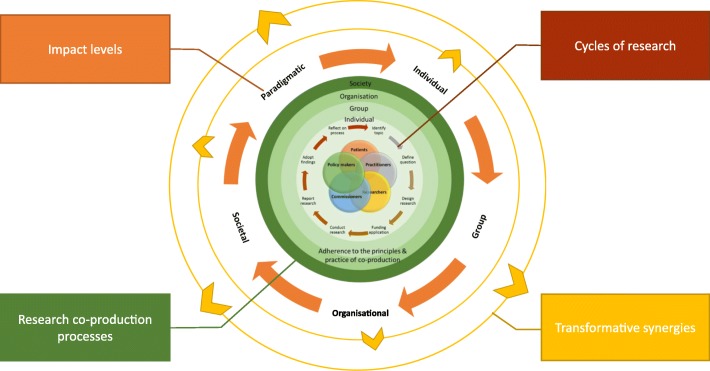
The transformative potential of co-produced research. This diagram shows how research co-production may engender impact at and across different levels (individual, group, organisational, societal, paradigmatic). These impacts are not finite, narrow or linear but broad, inclusive and dynamic. They have potential to initiate transformative synergies at a macro level, where they combine with other interventions, wider policies or practice and research priorities. These impacts are likely to include spin off research and increased capacity for research, ‘research stages’ are therefore illustrated as circular in this diagram rather than linear (as in Fig. 1). The degree of impact and potential to engender transformative synergism can be influenced by the co-produced projects’ placement on the research co-production continuum. For example: research studies, which successfully adhere to the principles and practice of co-production at all research stages, are large scale and involve multiple stakeholders, may realise greater impact at all levels and feed into synergistic change

The insights drawn from the literature, our case studies and workshops show how research co-production engenders change within, during and beyond the research project as a result of multiple social processes and productive interactions; it is dynamic and cyclical rather than linear and finite. These changes can be subtle and covert, starting at the micro-level but combining to seed macro-level change and the emergence of new ideas. These in turn may lead to transformative synergies [53] at a broader macro scale where co-produced research combines with other interventions, wider policies or practice priorities to create dynamic synergies. For example, micro actions by stakeholders within co-produced research may produce ‘self-organising’ macro-level changes, as exemplified in Case Study 5 (Additional file 5), where co-produced indicators had a national influence, or researchers may involve policy-makers to lever changes (e.g. Case Study 1 (Additional file 1), where national policy was altered, having a subsequent national impact [54]. Understanding interactions across different individual policy levels over time can help us reflect on what has changed, why and how. These reflections may then help feedback learning into future collaborations. However, the framework does not advocate any particular measurement instrument as impacts can be diverse, unpredictable, occur at different levels and be tangible or intangible. We propose that the cumulative effect of micro to macro multi-layered impacts of co-produced research can potentially lead to a virtuous cycle in which broader and more enduring transformation can occur (Fig. 2).

#### Case studies: factors that facilitated or challenged research co-production

By applying our framework to the six case studies we were also able to discern a range of factors that facilitated or hindered co-production. The collaborations and impacts described in our case studies did not emerge from a vacuum, participants needed time to learn, develop networks and trust. Our case studies’ life cycle started from an explicit position on co-production, collaboration, knowledge and implementation. While these case studies suggest significant and wide-ranging impacts from co-produced research (Boxes 3 and 4 and Table 3), our discussions and analysis also discerned key elements, activities and mechanisms that were commonly noted within our case studies as being essential to their achievement. Some of these elements appeared stable regardless of collaborator type, while others were affected by the context of the collaborator. These findings support the literature but also extend current knowledge by identifying those which facilitated co-production at specific levels (Table 4).

**Table 4:** Facilitators to co-production and achieving impact at each level

Level	Key elements, activities and mechanisms
Individual (micro)	• Regular interaction and communication between all parties • Keeping all parties on track and involved • Appreciative facilitation techniques • Trust, respect and openness • Being flexible and accommodating diversity of views • Reflexivity concerning one’s own values and social position, considering how to facilitate more equal relations with stakeholders who may hold less powerful positions
Group/interpersonal (micro)	• Defining roles and partnership infrastructure in large scale projects • In smaller ones, fluid and flexible relationships can work • Use of social media and information technology • Sustained supportive relationships • Regular meetings (face-to-face or web-based) • Facilitation and proactive management of potential power imbalances • Involving all parties in iterative cycles of data analysis • Flexibility to allow others to lead and suggest alternative routes • Ability to share knowledge, be open to others’ expertise and to admit gaps in one’s own • Core team members with boundary spanning and co-ordinating experience/roles
Organisational (meso)	• Scale, size and scope of project clearly defined and suited to the project question and team • Use of software to permit collaborative development of case study materials/outputs • Use of iterative dynamic and flexible processes that are responsive to contextual challenges and changing circumstances • Relevance and significance of the work to key stakeholders • Mechanism/integrated knowledge translation process or impact • Shared ownership, power and control of research study design, aims and outcomes • Involvement of experienced boundary spanners or individuals with dual clinical/academic roles
Societal (macro)	• Presenting information in engaging, accessible and creative forms, e.g. stories and film • Inclusion of authority figures/decision-makers • Use of more diverse, creative and/or accessible means of research dissemination
Paradigmatic (macro)	• Adherence to the principles and practice of co-production • Maintaining networks, brokering relationships and engaging with opportunities that arise from co-produced research • Wide and diverse dissemination of research outputs and methods • Advancing the practice, promotion and impact assessment of co-produced research

Our collective experience as researchers engaged in co-production (including our case studies) also highlighted challenges related to the process of working collaboratively. We found reconciling different stakeholder agendas and expectations and keeping projects within their scope could be difficult. Funders need to be aware that approaching research in this way requires additional resources (e.g. time to develop participant capabilities, funding for staff time to participate in research or backfill). Finding existing research evidence for the topic (e.g. originating from a practice/policy-making priority) can also be problematic, as relevant research may not be available [55]. Common challenges were maintaining practitioner engagement, maintaining project relevance in the face of constantly changing practitioner and policy-maker priorities, balancing this with service provision demands, co-ordinating multiple ethics applications, meaningful data analysis and interpretation by multiple stakeholders. Inter-agency or institutional data sharing can present issues, especially with different IT systems and stances on data confidentiality and security. Co-production partners in a number of our case studies also expressed concern at their ability to maintain momentum and dedicate sufficient time to prioritise this work, especially after the project ended.

### Strengths and limitations of our approach

Some members of the author team were known to each other before we set out to develop this paper and some were not; this ensured a wide spread of experiences, views and lively debate. Our choice of approach involved ‘walking the co-production walk and talking the talk’, meaning that it required time to understand each other’s positions, discuss ideas and gain consensus on our thoughts. Our ability to track, trace and capture multi-level impacts within and beyond our case studies was made possible by ongoing relationships nurtured in the co-production process.

We are all researchers (although KB and AlM also have clinical backgrounds) and our insights, though varied, all represent the researcher voice. We verified our case study summaries and impact grids with key co-production collaborators, but they did not contribute to this paper; thus, our inferences and conclusions may have benefited from these perspectives. In selecting our case studies, we gravitated towards co-produced research projects that had gone well, as these were more likely to generate micro to macro level impacts. This facilitated the development of the research co-production continuum and impact analysis framework. However, our choices were also informed by the techniques and philosophy of appreciative inquiry [56]. Additional insights may have emerged from reflection on negative cases. Further framework development and application will need to include a more systematic examination of the negative instances of impact. Our case studies focus on topics that were amenable to and benefited from co-production; not all healthcare questions can be answered in this way. Finally, while service users were involved as ‘participants’ in three case studies and co-production ‘contributors’ in another three (mainly at later stages of the research cycle, e.g. intervention development), they were not involved in co-production at earlier stages or throughout the research cycle.

### Recommendations and questions for future research

This concept paper proposes a continuum of research co-production, a social model of impact and a new framework for capturing the multi-layered impacts of this type of research. We offer it as a stimulus for debate, discussion and further research. The recommendations and research questions in Box 5 below are offered for research funders, policy-makers, managers and stakeholders involved in the co-production of knowledge and its application.

## Conclusion

History suggests research methods that explicitly aim to control and reduce complexity and contextual uncertainty and employ linear methods with the purpose of generating objective facts need to be balanced with other rigorous approaches to generating knowledge to inform healthcare quality and efficacy in the real world. The principles of co-production embrace complexity and uncertainty, potentially leading to a virtuous cycle of research processes and micro to macro level impacts with the ability not only to generate useful knowledge, but also to transform it into usable knowledge and to broaden research capacity in the process. Within complex human systems, emphases on the economic impact or end-of-project research outputs neglect the potential for the research process and productive human interactions to affect much deeper and more enduring change; our social model of impact aims to address this gap.

Co-production is challenging; it demands flexibility, reflexivity and boundary crossing, but when it works it results in insights and actions far greater than the sum of its contributory parts. Co-production can actively support the democratisation of knowledge and incorporate and blur the boundaries between different forms and sources of knowledge. It can provide the rich evidence required for effective policy and practice and foster ‘contextually adroit’ research-informed decision-making [14]. This may lead to more sustainable and wider impacts from intellectual and economic investment in research.

### Addendum

Following the initial phase of framework development described in this article, the authors presented and tested it further at a United Kingdom KM (http://knowledgemobilisation.net/) Forum 2018, workshop held in Bristol, United Kingdom. At this event, the authors facilitated workshop attendees in applying the framework to their own co-produced research, including projects where co-production was deemed to have been successful or those perceived of as having failed in some respect. This experience highlighted the need for guidance to assist others in using and testing it, which we subsequently developed (Additional file 8). This guidance is offered here as a preliminary means for co-production collaborators to operationalise the framework and capture impacts of their co-produced research. The authors anticipate that future work is likely to include further development of a Social Impact Framework tool; we welcome feedback to assist us in making it workable and accessible.

Our experience at the United Kingdom KM workshop also suggested the framework is applicable and useful for capturing impacts of projects where co-production was less successful, and/or the challenges involved impeded its completion or success. In one group discussion, they found that, by using the framework to reflect on micro-macro levels processes, impacts and mechanisms within a project that had been perceived as failing to achieve the expected outcomes, multiple impacts had actually occurred at all the levels, although they were not necessarily those initially anticipated or sought. Some of these impacts were significant and positive, especially at individual level, and had not been captured, or considered, before. The framework supported reflection on what had occurred, and highlighted that co-production had exerted a dynamic effect, akin to the scattering of billiard balls, and appeared to set in motion a range of unexpected processes and impacts. This warrants further investigation.

## Box 1 Knowledge mobilisation archetypes A, F & G from Davies et al. [3]

➢ Archetype A represents knowledge as a ‘research-based knowledge product’, produced and developed in universities and then ‘transferred’ through a linear process into policy and practice contexts, where knowledge users may (or may not) adopt the ‘knowledge product’

➢ Archetype F focusses on local learning and ‘absorptive’ capacity-building. Emphasises the co-production of knowledge generated locally within its context of use to aid effective mobilisation and implementation and is directed towards a wide range of outcomes

➢ Archetype G acknowledges the way in which research-based knowledge is transformed and moulded by encounters with different forms of knowledge and political and social forces. Archetype G activities therefore seek to develop and shape collaborations and networks to share expertise and increase their exposure to research knowledge [1]

## Box 2 Case studies included in our analysis, with references to associated publications

1. Developing evidence-enriched practice in health and social care with older people (CS1) [57–60]

2. What are the best indicators that public health agencies can use to monitor and guide their work in addressing the social determinants of health (CS2) [61–63]

3. Renewal of public health services in two provinces (CS3) [64–68]

4. A road less travelled: mapping children’s and families’ emotional journey following moderate to severe burn injury (CS4) (Paper under review)

5. Co-producing quality indicators for community nursing (CS5) [69, 70]

6. Proving the value of advice services (CS6) [71–73]

## Box 3 Specific level impacts. Note: individual level impacts are ordered from service user to researcher; however, impacts at other levels were more generic and are presented in no particular order. References in brackets indicate in which of the six case studies (Additional files 1, 2, 3, 4, 5 and 6) the impact occurred

### Individual level (micro)

Individual impacts of co-produced research varied according to the type of individual involved (e.g. service users, practitioners, researchers, managers, policy-makers). Common impacts included being heard, gaining confidence, networks and skills, and increased engagement with future research

Additional impacts included:

- Service users were more engaged in routine care, some developed additional creative research outputs with help from members of the team (e.g. a booklet and CD) (CS1)
- Practitioners reported increased job satisfaction and became more reflective of their practice (CS1, CS4)
- Some practitioners took steps to advance their research careers (CS3, CS4, CS5, CS6)
- Researchers developed boundary spanning skills leading to spin-off research and knowledge mobilisation careers (CS4, CS5, CS3)
- Collaborations increased researchers’ ability to conduct their research with vulnerable groups (CS1, CS4, CS6)

### Group level (micro)

Note: the following group level impacts were noted in ALL, or some, of our case studies (as indicated in the brackets); however, the degree and manifestation of these impacts varied by the type of groups involved

- Improved understanding and acceptance of each other’s worlds and lived experience (ALL); this impact also occurred at individual level
- Increased trust and willingness to work together in the future (ALL)
- Transfer, exchange and recognition of complementary knowledge and skills (ALL)
- Improved networking and communication between all parties (ALL)
- Some relationships led to further collaborative research between researchers and practice partners (CS1, CS3, CS4, CS5)
- Interactions within groups, e.g. service users enabled to share stories, exchange contact details and feel less alone (CS1, CS4)
- Interpersonal relationships between members of the core study team enabled an effective response to a funding crisis (CS6)

### Organisational level (meso)

Note: the following organisational level impacts were noted in ALL, or some, of our case studies (as indicated in the brackets); however, the degree and manifestation of these impacts varied by the type of organisation involved

- Organisational capacity-building through sharing knowledge and skills (ALL)
- Developing organisational competency and confidence in research and practice (ALL)
- Organisations securing further research funding and inspiring spin-off ideas (ALL)
- More intricate, contextually informed analysis leading to implementable outcomes due to stakeholder buy-in (ALL)
- Increased competence and sensitivity towards the culture, contexts and challenges of other stakeholders’ worlds (ALL)
- Output integrated into university clinical training module for medics and nurses (CS4)
- Delivery of tangible outputs such as papers, conference presentations, practice and/or policy change (ALL)
- Added value to participating organisations (ALL), CS1 may be returned as a Research Excellence Framework ‘case study’
- Raised awareness of ‘public health systems and services research’ as an emerging research area (CS3)

### Societal level (macro)

- Health quality equity indicators now used by many of the 36 health units in Ontario, Canada, to evaluate their own health equity work (CS2)
- Project approach woven into the National Dementia Learning and Development Framework for Wales (CS1)
- Evidence to funders that their commissioned research and policy direction can be implemented into practice within service developments; showcasing of funders’ commissioned research (CS1, CS4)
- National voluntary sector organisations or service providers altered policy across all provision (CS1)
- Policy brief developed and disseminated by the Institute for Policy Research at the University of Bath and local media coverage. Research findings were published in national media and a UN call for evidence on extreme poverty and human rights, providing evidence of the adverse impact of welfare reform in the United Kingdom (CS6)
- Uptake of new approaches to monitoring, evaluating and guiding progress towards population wellbeing and prevention work (CS1, CS6)
- Wider adoption of research-based indicators, e.g. integrated into practice by another healthcare provider, which later was awarded the performance assessment of ‘outstanding’ (CS5)
- Uptake of a story-telling approach ‘Most Significant Change technique’ to monitoring and evaluating well-being and prevention work across two Welsh local authorities (CS1)
- Independent quality regulators, e.g. the United Kingdom care quality commission, which considered the incorporation of several of the community nursing quality indicators into their national scheme (CS5)
- Conference presentations and journal articles (ALL), showcasing diverse stakeholder voices using innovative formats (CS4)
- Proliferation of new conceptual approaches, e.g. knowledge mobilisation and co-production (ALL)
- Initiated constitution of a United Kingdom national (England, Scotland, Wales) academic and practitioner narrative and dialogue-based research and practice development group (CS1)

## Box 4 Broad impacts occurring at all levels

### Knowledge required for effective healthcare policy and practice

- Co-production acknowledged, harnessed and perpetuated the democratisation of knowledge (through increasing understanding of other perspectives, engagement and inclusiveness)
- Patient, practitioner, policy-maker and manager stories, experiences and contextual knowledge were woven into research processes, policy and stakeholder institutions thus bringing complex human and contextual realities within healthcare to the fore
- Knowledge combinations optimised the potential for research knowledge to be transformed into knowledge-in-practice-in-context [1]

### Research for healthcare policy and practice

- Led to relevant research with significance for policy and practice
- Put less frequently heard stakeholders, and relational and situational factors at the heart of research
- Encouraged cross fertilisation of ideas
- Enabled research to happen, through contributions of resources, introductions to other gatekeepers in the system, or by working with especially hard-to-reach participants
- Necessitated development of more agile, flexible research processes to adapt to changing contexts and needs

### Capacity for research

- Increased and diversified the sphere in which research is understood, generated and used
- Developed bridge-building and boundary spanning individuals and knowledge mobilisation knowledge and skills
- Created enduring relationships and networks
- Led to spin-off research and added impetus to development of new research or joint clinical/research careers
- Optimised skill sharing and efficiency through partnering and collaboration
- Created direct links between practice and research, and in some cases, policy
- Broke down barriers and enabled boundaries to be crossed
- Created opportunities for serendipitous productive encounters
- Addressed and confronted power imbalances between research users, generators and recipients
- Created opportunities for the development of more heterogeneous multifaceted ‘communities of practice’

### Nature of impact

- Generated a diversity of dissemination approaches
- Enabled transformations in care and policy and of research approaches
- Led to implementable, contextually informed outcomes, and diverse accessible outputs
- Increased the likelihood of unexpected outputs

## Box 5 Recommendations and research questions arising from this paper

Recommendations:

- Impact assessment needs to be expanded to emphasise and reward the often hidden social and transformational effects that co-produced research may generate
- Impact measures need to capture micro to macro level impacts – they need to include those which happen within and beyond the research process (as a result of productive interactions) as well as those directly related to research results
- More needs to be known about what makes co-produced research successful (or not); those using (and evaluating) co-production approaches could build in more time to determine what it is that works and why, thereby extending the knowledge base about co-produced research
- Impacts may manifest several years after collaborative research; this analytic framework may help researchers reflect on what has catalysed impacts over time, and why
- Our analytic framework needs further development; research co-producers (from all stakeholder groups) seeking to capture the breadth of their impact might apply and test the framework’s applicability to their work
- Teams undertaking co-produced research might consider implementing means to continuously map and review impacts during and beyond project completion; these could be based on our framework. This would clearly have funding and time implications but would provide a more accurate picture of impacts as they emerge in real time
- Funding for co-produced research needs to account for the additional time required to successfully execute and evaluate this approach

Research questions:

- What types of impact (outputs, uses, outcomes) does co-production optimise and how?
- How does a ‘social model of impact’ enhance our thinking about (and actions around) impact?
- How can impacts, including unintended ones, from research co-production be determined over time?
- Which co-production mechanisms are likely to engender impact and lead to transformative synergies?
- What are the possible negative consequences/impacts and challenges of co-production? How can this ‘dark side’ of co-production [29] be ameliorated?
- What are the relationships between the different elements of the research co-production continuum (research stages, types of contributor, scale of contribution, and adherence to co-production principles)? How do essential factors such as key individuals’ leadership approaches and stakeholder engagement affect co-production processes and research impact?
- How can current impact indicators and metrics, such as those developed by Barwick [43, 44], be built into this social impact model?
- What are the specific benefits, challenges and impacts of co-production involving service users throughout the research cycle?
- What are the paradigmatic implications of co-production and how does this worldview fit with other research paradigms?

## Additional files

Additional file 1: Case study 1. (DOCX 18 kb)

Additional file 2: Case study 2. (DOCX 17 kb)

Additional file 3: Case study 3. (DOCX 18 kb)

Additional file 4: Case study 4. (DOCX 19 kb)

Additional file 5: Case study 5. (DOCX 18 kb)

Additional file 6: Case study 6. (DOCX 22 kb)

Additional file 7: Case analysis grid. (DOCX 15 kb)

Additional file 8: Guidance for using the Social Impact Framework. (PPTX 80 kb)

## Abbreviations

IKT: Integrated Knowledge Translation
KM: knowledge mobilisation
