# Supplementary material for: Embracing complexity and uncertainty to create impact: exploring the processes and transformative potential of co-produced research through development of a social impact model
Source: Health Res Policy Syst. 2018 Dec 11;16:118. doi: 10.1186/s12961-018-0375-0 (PMC6288891; doi:10.1186/s12961-018-0375-0)
Supplement: Supplementary file 1 — Case study 1. (DOCX 18 kb) [file 12961_2018_375_MOESM1_ESM.docx]

**Case study 1 (CS1)** Title: Developing Evidence-Enriched Practice in Health and Social Care with Older People

**Funder:** Joseph Rowntree Foundation (JRF), UK

**Co-producers:** Project co-design and conduct: JRF project manager and multidisciplinary research team including social workers, NHS practitioners and researchers. Additional practitioners, service users and carers involved in co-design of interventions. **Project lead:** University-based PI.

**Aim:** To tackle two central issues inhibiting improvements in social care for older people namely: limitations to the use of evidence in social care and prevailing negative discourse associated with older age. To use conversational inclusive action research methods and diverse forms of evidence including the JRF programme ‘A Better Life’ to improve social care user and provider research engagement and enhance older peoples’ quality of life.

**Method:** The JRF programme ‘A Better Life’ generated comprehensive evidence about what wellbeing means for older people and their carers; this was encapsulated in seven challenges. The All Wales Academic Social Care Research Collaboration (ASCC) and the Institute for Research and Innovation in Social Services (IRISS) formed a partnership with JRF to explore and develop an approach to using this evidence to develop services/the workforce considering the complexities involved. The project used a collaborative action research design involving older people, carers, researchers, managers and frontline staff from social care and health organisations, both statutory and voluntary. Each of 6 research sites chose specific topics for service and workforce development, including; relationship centred practice, positive and rights-based approaches to risk management, the development of meaningful activities, short breaks, addressing loneliness and better recording and paperwork. Diverse partners were involved in a range of co-production activities to help participants engage with the evidence (including research findings transformed into engaging summaries and stories told from older people, carer and staff perspectives), define priorities for service improvements and plan how to achieve them.

**Scale:** The project ran for one year in six sites, five in Wales and one in Scotland;

**Impact/outcomes -** this project led to diverse and wide influence/impact beyond these 6 sites (see below).

1. Individual

***Older people and their carers;*** improved wellbeing, increased involvement in routines of care. Creative outputs developed by service users e.g. book about bullying and a musical CD featuring a care home resident.

***Social and healthcare practitioners:*** Those involved in the research co-design and conduct gained experience in research and co-production techniques; they widened their networks leading to spin off research. Practitioners involved in ‘intervention’ co-design experienced increased job-satisfaction, gained awareness of practical implementation problems and swapped ideas for better practice. They gained insight into the processes and funding of research, the work of JRF and ‘lived experience’ of old age.

***Researchers*** Gained experience in health & social care sector engagement and the use of co-production methods. One subsequently moved into a full-time university positions bridging academic and practice worlds.

***JRF*** project manager responsible for a successful completed research study with significant research, practice, and policy impacts. Direct contact between research funder, participants and policy makers

1. Interpersonal & organisational

Participants across all the sites reported enhanced wellbeing due to their involvement, indicating development of an ‘enriched environment’ of learning (Nolan et al 2006). Participants felt a sense of security, continuity, belonging, purpose, achievement, and significance – that they mattered, and that things could change for the better. The evaluation revealed improved relationships, greater networking opportunities, information exchange and increased trust among professionals and between policy makers, managers, professionals, older people, carers and different sites. Secured further University funding for practice development, will inform an Impact Case study for inclusion in next UK Research Excellence Framework assessment. Publications (3) and wide dissemination via numerous conferences. Linkage with other networks and universities. Practice changes (all sites). Multiple alterations to delivery/content of staff education/development

1. Societal

Demonstrated the success of co-production and appreciative facilitation techniques. Illustrated the necessity for diverse evidence and innovative inclusive approaches to its use. Changes in organisational policy across all of one provider’s services based on clear but flexible notions of professional boundaries (‘Professional Boundaries’ policy replaced with a ‘Shared Lives and Professional Boundaries’ policy). Uptake of a story telling approach ‘Most Significant Change technique’ to monitoring and evaluating well-being and prevention work across two Welsh local authorities. Project approach woven into the national Dementia learning and development framework for Wales. Initiated constitution of a UK national (England, Scotland, Wales) academic & practitioner narrative and dialogue-based research and practice development group.
