# Supplementary material for: Embracing complexity and uncertainty to create impact: exploring the processes and transformative potential of co-produced research through development of a social impact model
Source: Health Res Policy Syst. 2018 Dec 11;16:118. doi: 10.1186/s12961-018-0375-0 (PMC6288891; doi:10.1186/s12961-018-0375-0)
Supplement: Supplementary file 2 — Case study 2. (DOCX 17 kb) [file 12961_2018_375_MOESM2_ESM.docx]

**Case study 2 (CS2)** Title: What are the best indicators that Ontario Public Health agencies can use to monitor and guide their work in addressing the social determinants of health

**Funder:** Public Health Ontario, Canada

**Co-producers:** Project design and conduct: frontline public health staff, medical officer of health and academics from 2 universities (including 2 students); in addition, staff at four public health units were involved as participants in the design and testing of organizational indicators. **Project lead:** Medical Officer of Health

**Aim:** 1) to identify a comprehensive set of indicators that boards of health could use to monitor and guide progress toward fulfilment of public health roles to address the social determinants of health and reduce health inequities, 2) to establish face validity of the indicators, according to inequity experts, 3) to test the indicators for feasibility, understandability, relevance, validity, reliability and comparability. A further aim of the funding call was to build research capacity among public health units and support stakeholder partnerships

**Method:**

This two-year Integrated Knowledge Translation project was accomplished through literature review & key informant interviews; the development and refinement of indicators, pilot testing of indicators with four health units; and the revision of indicators. Two graduate student research assistants were hired to help with the work. Regular web/teleconferences were scheduled with the full research team, while smaller sub-groups of researchers and practitioners worked between the meetings to execute aspects of the research. To facilitate even more involvement, a web-based software platform that allowed users to make changes and view others’ changes on documents, much like Google docs, was used when it came time to develop the indicators. Practitioners identified the first draft of the research questions, then invited academics to join the research team. Everyone developed the proposal, wrote relevant ethics board applications, assisted with the research process, interpreted the findings, and co-authored the final reports and presentations. The team worked through a dynamic, iterative process to produce a comprehensive set of evidence informed contextually relevant indicators to be tested.

**Scale:** This project involved two universities and five Ontario-based Public Health Units

**Impact/outcomes -** this project lead to diverse and wide influence/impact (see below).

1. Individual

***Public Health Unit staff:*** a) research participants at pilot sites of indicator testing confirmed some of their health equity related activities were ‘on the mark’ and raised awareness of what further work they could be doing in terms of health equity, b) practitioner members of the research team gained research experience and skills in literature review, key informant interviews, development and piloting of indicators, and development of research outputs. c) student nurse research assistant also gained applied research experience and skills.

***University Academics:*** *a)* academic members of the research team gained experience conducting IKT research within the public health sector*,* and understanding the political circumstances of public health services. b) student graduate research assistant also gained applied research experience and skills.

1. Interpersonal & organisational**:** University and health unit based team members contributed equally to the knowledge generation and dissemination in an Integrated Knowledge Translation fashion. Capacity for future work in this area was developed. In some health units the work raised awareness of what further health equity activities they could be doing. One manuscript is published, the other is under review.
2. Societal. Many of the 36 health units in Ontario are now using the health equity indicators in some fashion (adapted for local context etc.) to evaluate their own health equity work. This project has been widely disseminated to diverse audiences through varied means including workshops, conferences and practitioner training webinar. A website and online host has been developed with indicator information, worksheets and data and reporting applications that will allow users to save and track their HE activity progress over time.
