# Supplementary material for: Embracing complexity and uncertainty to create impact: exploring the processes and transformative potential of co-produced research through development of a social impact model
Source: Health Res Policy Syst. 2018 Dec 11;16:118. doi: 10.1186/s12961-018-0375-0 (PMC6288891; doi:10.1186/s12961-018-0375-0)
Supplement: Supplementary file 3 — Case study 3. (DOCX 18 kb) [file 12961_2018_375_MOESM3_ESM.docx]

**Case study 3 (CS3)** Title: REPHS study: Renewal of public health services in Ontario (ON) and British Columbia (BC)

**Funder:** Canadian Institutes for Health Research (CIHR), Canada

**Co-producers:** Co-design and conduct of research: Public health academics, policymakers and frontline public health staff (including managers and senior administrators) from ON & BC. **Project lead:** Academics and Policymaker Knowledge User

**Aim:** The purpose of this five-year program of research is to conduct a set of studies that compare the implementation and impact of the BC Core Public Health Functions framework and the Ontario Public Health Standards. Overarching goals of this research program were to: a) to inform public health systems renewal in BC and ON (and in Canada) and, in turn, contribute to improving population health and reducing health inequities; b) advance the field of public health services research in Canada by implementing a consensus-based research agenda and applying/developing innovative research methodologies; c) inform integration and linkage of public/population health and primary care services; and d) train expert public health services and population health researchers.

**Method:** The research questions were conceptualized within a case study design, in which each case was defined as the core public health program. Collaborators in each province collectively defined and refined the research questions, these were then reconciled and consensually agreed across both provinces. Documents were collaboratively written to outline how project activities would proceed, including an Authorship Agreement, a Values Statement that was inclusive of all types of knowledge and research approaches, and a Student Policy to ensure that student involvement was consistent and fair. These documents took about a year to develop collaboratively. Research methods and PH practitioner knowledge of local context and constraints were iteratively woven into the research process by regular face-to-face/online communication and use of mutually accessible formats for sharing information. This research program applied complex adaptive systems theory, which highlights the importance of context in understanding the implementation and impact of complex public health systems interventions. Each province had a sub-team to carry out the research. The main data collection and analysis strategies included: focus groups, key informant interviews, document reviews, secondary data analysis, concept mapping, and social network analysis. A governance structure was established to ensure each province, which organised public health services quite differently, could move forward with the research process in a locally relevant way while maintaining consistency across provinces.

**Scale:** Large scale complex research program across two Canadian provinces

**Impact/outcomes -** this project lead to diverse and wide influence/impact (see below).

1. Individual

***Public Health Unit staff and Academics:*** The entire research team comprising 12 public health units (i.e., front line PH staff) from ON and BC, about 6 public heath government officials from ON and BC, several academics from various universities, and student research assistants gained IKT research experience in the public health context. At least one government official went back to school for graduate studies.

2. Interpersonal & organisational**:** A number of co-authored publications and presentations were disseminated over the five years, and publications continue to be written. Academics wrote spin-off grants based on the strong partnership; important public health networks were developed. All participants raised awareness of the project within their own organizations; the team was invited to present the work to wider government audiences. Overall the team established a high level of credibility among the public health community in both provinces.

3. Societal. This work raised awareness of ‘public health systems and services research’ as an emerging research area; one of the PIs contributed to an editorial about the importance of the research area in Canadian Journal of Public Health (i.e., system awareness starting to happen). The research influenced the revision of some public health policies in each province.
