# Supplementary material for: Embracing complexity and uncertainty to create impact: exploring the processes and transformative potential of co-produced research through development of a social impact model
Source: Health Res Policy Syst. 2018 Dec 11;16:118. doi: 10.1186/s12961-018-0375-0 (PMC6288891; doi:10.1186/s12961-018-0375-0)
Supplement: Supplementary file 4 — Case study 4. (DOCX 19 kb) [file 12961_2018_375_MOESM4_ESM.docx]

**Case study 4 (CS4)** Title ‘A road less travelled: mapping children’s and families emotional journey following moderate to severe burn injury’

**Funder:** [Roald Dahl Marvellous Children’s charity](http://www.roalddahl.com/charity) – ‘Marvellous Nurse Inventing Room’ grant, UK

**Co-producers:** project co-design and conduct: University Hospitals Bristol Paediatric Burns service/[Scar Free Foundation](http://scarfree.org.uk/) Research Nurses, University of the West of England Research Fellow, Burns service clinical psychologist and clinical nurse specialist. Co-design event: Families and children who had experienced a moderate or significant burn injury. Project lead: Scar Free Foundation Lead Research Nurse

**Aim:** 1) To map key trigger points (emotionally significant) in the families/child’s psychological journey after a significant burn injury to inform targeted service improvements, 2) to enhance capacity for nurse led research

**Method:** This project followed an abbreviated form of ‘Experience based co-design’ as described by the ‘Kings Fund’ online toolkit, now available online from the [Point of Care Foundation](https://www.pointofcarefoundation.org.uk/resource/experience-based-co-design-ebcd-toolkit/). It involved the following stages: 1) in-depth exploration of key stages and trigger points in the emotional journey following a child’s moderate to severe burn injury - from family and practitioner perspectives; 2) production of a short-edited film illustrating the family experience (derived from thematic analysis of filmed parent interviews - as the children were generally under 5 years of age), 3) design of a poster illustrating practitioner perspectives on the same journey informed by thematic analysis of recorded interviews, 4) key stakeholder (research involved and wider families, practitioners and researchers) co-design event to watch and discuss the film/poster, identify key areas for service improvement/implementable changes in practice and agree next steps.

**Scale:** A small scale project aimed at improving local services and research capacity.

**Impact/outcomes -** this project lead to influence/impact beyond the local area (see below)

#### Individual

***Family outcomes:*** Parents attending the co-design event shared stories, drew emotional support from each other and swapped contact details. They expressed surprise that this was ‘research,’ all subsequently signed up to be involved in future burns research, one is now named on a large programme grant. The co-design event created a safe environment for extremely young children with burns, and siblings, to swap stories and have their voices heard. Families of children who had experienced a more recent burn found the film helped them in anticipating future challenges and suggested its use as a family information/education tool.

***Practitioner outcomes:*** Increased capacity for nurse led research resulting in further collaborative research bids. Supported experience in using diverse and/or new research skills e.g. thematic analysis of filmed material. Increased awareness of key areas for practice improvement and the family experience of care; this initiated discussion of wider use of the film as a clinical training tool. Diverse opportunities to disseminate a distinct creative approach to research and for practitioner contextual/clinical knowledge to be valued/heard.

***Researcher outcomes:*** The researcher who had encountered EBCD as a potential co-production method was able to experience its application to a real-life problem; this informed a successful NIHR Knowledge Mobilisation Research Fellowship application.

1. *Interpersonal & organisational*

All collaborators experienced the conduct and potential of co-produced research, developed knowledge, skills and new networks. Evaluation of the co-design event showed all participants felt their voice mattered, were more likely to engage in future research and found sharing stories, ideas and perspectives simultaneously challenging, poignant and extremely useful. Tangible outputs include: a 25-minute film illustrating stages and key trigger points in the first year (available to all Trust staff). Poster illustrating practitioner perspectives on the same topic. Peer reviewed collaborative article(s) under development. Initiatives to target identified areas for service improvement have been put in place through; implementable modifications in practice, development of new materials (e.g. a patient held diary); or are the subject of further research. The hospital trust Patient Experience lead has expressed interest in wider local use of EBCD to improve services. University and healthcare institutions have both benefitted from increased capacity and funding for applied research.

#### Societal

Project findings, film (and method) have been disseminated to multiple academic, practitioner and policy maker audiences at local, national and international conference/events. These have demonstrated the strengths of EBCD/co-produced research, emphasised the significance of experiential aspects of care and led to many enquiries from within practice and research. The film has been extremely well received by diverse academic and practitioner audiences. They have been visibly moved and commented on its ability to evoke a rational and emotional response and create a compelling argument for change (e.g. an NHS England representative commented *‘would value having this as a resource to use’*. The film has been integrated into a ‘psychosocial care’ training module for nurses and medics, potential revisions for its use as a tool for future parents are under discussion.
