# Supplementary material for: Embracing complexity and uncertainty to create impact: exploring the processes and transformative potential of co-produced research through development of a social impact model
Source: Health Res Policy Syst. 2018 Dec 11;16:118. doi: 10.1186/s12961-018-0375-0 (PMC6288891; doi:10.1186/s12961-018-0375-0)
Supplement: Supplementary file 5 — Case study 5. (DOCX 18 kb) [file 12961_2018_375_MOESM5_ESM.docx]

**Case study 5 (CS5)** Title: Co-producing quality indicators for community nursing

**Funder:** This project was commissioned by a local healthcare policy-maker and was funded by Bristol Primary Care Trust, Bristol, UK

**Co-producers:** In Phase 1 a team comprising academics from the University of Bristol and University of the West of England were responsible for identifying relevant research for indicator development while community practitioners, service users and commissioners were involved as co-designers of indicators. Phase 2 involved two of the original academics, the community nursing lead and a community practitioner to test the indicators.

**Project lead:** Research fellow (academic) and community nursing lead (practice)

**Aim:** Phase 1: To devise quality indicators for community nursing. Phase 2: to test their acceptability and feasibility as community nursing quality indicators.

**Method:** In Phase 1 the university team ran a series of focus groups with community nurses, conducted service user, specialist nurse and commissioner interviews and reviewed research based standards and relevant research to comprehensively explore and capture dimensions of quality within community nursing practice. They employed iterative cycles of analysis and informant validation through further discussion with service users, community & specialist nurses and commissioners to define and refine potential standards. Community nurses had the ultimate say on which quality indicators to include (those selected were: wound, diabetes and end of life care and patient experience). In Phase 2 the quality indicators were tested through implementation within 12 community nursing teams.

**Scale:** The project involved academics at two Bristol Universities and multiple service provider and user groups

**Impact/outcomes -** this project lead to diverse and wide influence/impact. Significant impacts were delayed and occurred many years after the project end (see below)

1. Individual

***Phase 1 Service user & community and specialist practitioner participants:*** gained a better impression of research and willingness to work with researchers in the future

***Phase 1 & 2 Practitioners:*** gained confidence in research design, methods and dissemination. The community nursing lead subsequently published two professional journal articles and undertook a Master’s degree.

***Phase 1 & 2 Researchers:*** gained experience in the principles and practice of co-produced research: for one this informed development of a successful Knowledge Mobilisation Research fellowship application and the other researcher subsequently applied for and secured a large 500K follow on grant (her first as project lead)

2) Interpersonal & organisational

The project increased trust between contributors, improved networking and led to further collaborative research. Researchers benefitted from practitioner’s knowledge of research interested practitioners to contribute to future bids. One researcher supported the community nursing lead through her Master’s degree, recommended her for and mentored her through a Nurse leadership programme. Overall, the indicators were found to be acceptable and usefully measured key aspects of quality in community nursing. They highlighted areas of good practice which could be shared across the service and identified problematic areas. Moreover, the evaluation demonstrated an improvement in the standard of care. Two peer reviewed articles in scientific and professional journals were also published. However shortly after the feasibility study report was released, a collective decision was taken by the project team and senior healthcare managers to stop implementation because many other types of indicators were required nationally and locally. The quality indicator scheme was discontinued in light of increasing administrative burden.

1. Societal

The project had no impacts at this level until much later when individuals, organisations and events contributed to realising the following local and national impacts:

1. Delayed impacts

Management consultants on behalf of the Care Quality Commission (CQC), the body that inspects health and social care services nationally, read the Phase 1 scientific paper a few years later, contacted the researchers and suggested the incorporation of several of the quality indicators into the national scheme. Thus some indicators potentially had national reach. Moreover, in her new role as an end of life care facilitator in a different community healthcare provider the lead community nurse, developed care plans and protocols based on Phase 1 & 2 end of life care indicators and adapted patient reporting tools, which were incorporated into standard electronic systems locally. In a subsequent CQC inspection, this community healthcare provider was assessed as offering ‘outstanding’ end of life care because patient outcomes were routinely monitored and reported.
