# Supplementary material for: Embracing complexity and uncertainty to create impact: exploring the processes and transformative potential of co-produced research through development of a social impact model
Source: Health Res Policy Syst. 2018 Dec 11;16:118. doi: 10.1186/s12961-018-0375-0 (PMC6288891; doi:10.1186/s12961-018-0375-0)
Supplement: Supplementary file 6 — Case study 6. (DOCX 22 kb) [file 12961_2018_375_MOESM6_ESM.docx]

**Case study 6 (CS6)** Title: Proving the value of advice

**Funder:** The UK Big Lottery Fund Research programme [South West Forum Proving Our Value Programme](http://southwestforum.org.uk/?q=POV)

**Co-producers:** Project co-design and conduct: university staff, advice service staff and volunteers. Additional collaborators were: The South West Forum (SWF) Proving Our Value Programme; and a national advice service organisation impact and evaluation lead had intermittent involvement. Advice service clients were involved as research participants. **Project lead:** University of Bath

**Aim:** 1) To understand, measure and value the long term social and economic value of advice provided to clients by an advice service organisation and, 2) develop methods for evaluating the long term social impact of advice services.

**Method:** Research carried out by University staff, and trained advice service volunteers. Project management group included university staff, advice service staff and volunteers. Interviews and data analysis were conducted by research staff and advice service volunteers (trained, supervised and co-ordinated by University staff). Advisors supported recruitment of clients onto research; the trust developed between advisor and client was important to recruitment. Research interviews were conducted by trained advice service volunteers and University staff. Joint analysis by advice service research volunteers and University researchers supported understanding of the advice service process and managing large amounts of data. Halfway through the project, local government informed the advice service organisation about a proposed 55% reduction in funding due to cuts. Researchers worked with advice service staff to use emerging research findings to illustrate how advice services were contributing toward local government statutory targets.

**Scale:** This project involved one local advice service organisation and University. It formed one of five funded projects, all working collaboratively between third sector organisations and an academic partner to develop social impact methods, within South West England.

**Impact/outcomes:** The co-production of this research enabled it to be responsive to a rapidly changing context in which severe funding restrictions were threatened. The project delivered planned outcomes in terms of service evaluation, organisational capacity building, and evidence for funding debates.

1. Individual impacts

***Advice service research volunteers:*** developed research skills and experience.

***Advice service staff:*** supported in evidencing the impact of advice services.

***Researchers:*** developed skills in collaborative and co-produced research.

1. Interpersonal & organisational impacts

Research co-production enabled larger scale research project than would otherwise have been possible. [Full peer reviewed report](http://opus.bath.ac.uk/39052/5/SWF_Proving_our_Value_CAB_Bath_April_2014_1.pdf) openly published. Separate interim report written for local government based on emerging research findings to support the local organisation in evidencing impact. Practitioner events across the region developed by the SWF Proving our Value programme to share learning about social impact methods. Alongside other submissions, University staff presented findings at a council debate about advice services funding. This debate resulted in the Council developing an advice strategy and reviewing the proposed 55% budget cut. In the long-term, this was reduced. Research methods and questions were edited and expanded quantitatively, to inform a national ROI questionnaire, supporting wider national impact and evaluation work. After project end, lessons of co-production incorporated into further local impact work with different advice services.

1. Societal impacts

Members of research team interviewed on [local radio](http://www.bath.ac.uk/news/bathinpapers/index2014.shtml), research featured in [local paper](http://www.bath.ac.uk/news/bathinpapers/index2014.shtml). A [policy brief](http://www.bath.ac.uk/publications/proving-the-value-of-advice-a-study-of-the-impact-of-citizens-advice-bureau-cab-services/attachments/citizens-advice-bureau.pdf) developed and disseminated by the Institute for Policy Research at the University of Bath enabled different organisations and policy makers to access this research. Whilst intervention by the research team may have helped to secure local reductions in cuts, local advice service organisation funding, as elsewhere, is still under threat due to local government budget cuts. The research enabled regional and national capacity-building and scaling-up to evaluate the impact of advice. The research contributed to an academic critique of social return on investment (SROI) (where all social impacts are translated into economic values), illustrating the wider public value of advice services, including democratic values such as justice and equality. Research findings were published in [The Conversation](https://theconversation.com/advice-is-a-lifeline-for-people-claiming-benefits-but-support-services-are-under-threat-from-cutbacks-98893), and a [UN call for evidence](https://www.ohchr.org/EN/Issues/Poverty/Pages/UKVisitSubmissions.aspx) on extreme poverty and human rights, contributing to societal debates about the adverse impact of UK welfare reform.
