# Supplementary material for: Embracing complexity and uncertainty to create impact: exploring the processes and transformative potential of co-produced research through development of a social impact model
Source: Health Res Policy Syst. 2018 Dec 11;16:118. doi: 10.1186/s12961-018-0375-0 (PMC6288891; doi:10.1186/s12961-018-0375-0)
Supplement: Supplementary file 7 — Case analysis grid. (DOCX 15 kb) [file 12961_2018_375_MOESM7_ESM.docx]

**Supplementary Information File H**

**Framework for capturing multi-level processes, impacts and key mechanisms of co-produced research (example grid)**

| **Level** | **Research process** | **Impacts** | **Key mechanisms or elements** |
| --- | --- | --- | --- |
| *Individual(s)* | *People involved and how:* | *People impacted and how:* | *Factors which assisted research processes to occur and create impact at this level:* |
| *Group(s)/ interpersonal relations* | *Important interpersonal relations involved and how:* | *Impact of/on interpersonal relation (including partnerships, practice change within teams or departments):* | *Factors which assisted research processes to occur and create impact at this level:* |
| *Organisation(s)* | *Institutions involved and how:* | *Impacts on institutions and organisations (including structures, rules, norms, culture, changes in practice across organisations):* | *Factors which assisted research processes to occur and create impact at this level:* |
| *Society/*  *infrastructure* | *Infrastructural actors involved and how (e.g. thinktanks, policymakers):* | *Infrastructure impacted: impacts occurring at national or international level):* | *Factors which assisted research processes to occur and create impact at this level:* |
| *Paradigmatic* | **The process here is ‘adherence to the principles and practice of research co-production’** | *Impacts at a conceptual or theoretical level:* | *Factors which assisted research processes to occur and create impact at this level:* |
