# Supplementary material for: Embracing complexity and uncertainty to create impact: exploring the processes and transformative potential of co-produced research through development of a social impact model
Source: Health Res Policy Syst. 2018 Dec 11;16:118. doi: 10.1186/s12961-018-0375-0 (PMC6288891; doi:10.1186/s12961-018-0375-0)
Supplement: Supplementary file 8 — Guidance for using the Social Impact Framework. (PPTX 80 kb) [file 12961_2018_375_MOESM8_ESM.pptx]

## Slide 1
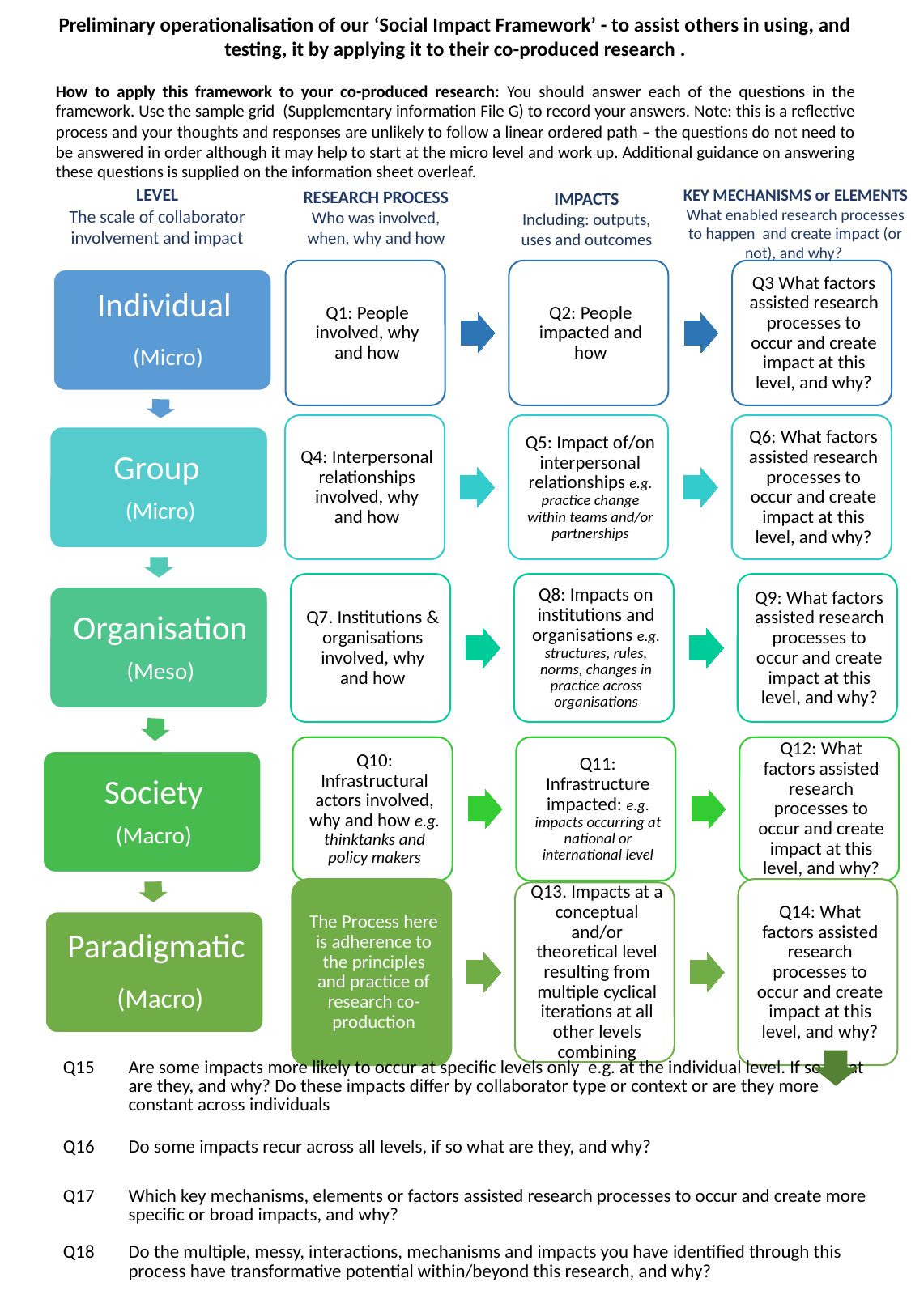

Preliminary operationalisation of our ‘Social Impact Framework’ - to assist others in using, and testing, it by applying it to their co-produced research .
How to apply this framework to your co-produced research: You should answer each of the questions in the framework. Use the sample grid (Supplementary information File G) to record your answers. Note: this is a reflective process and your thoughts and responses are unlikely to follow a linear ordered path – the questions do not need to be answered in order although it may help to start at the micro level and work up. Additional guidance on answering these questions is supplied on the information sheet overleaf.
LEVEL
The scale of collaborator involvement and impact
RESEARCH PROCESS
Who was involved, when, why and how
IMPACTS
Including: outputs, uses and outcomes
KEY MECHANISMS or ELEMENTS
What enabled research processes to happen and create impact (or not), and why?
| Q15 | Are some impacts more likely to occur at specific levels only e.g. at the individual level. If so what are they, and why? Do these impacts differ by collaborator type or context or are they more constant across individuals |
| --- | --- |
| Q16 | Do some impacts recur across all levels, if so what are they, and why? |
| Q17 | Which key mechanisms, elements or factors assisted research processes to occur and create more specific or broad impacts, and why? |
| Q18 | Do the multiple, messy, interactions, mechanisms and impacts you have identified through this process have transformative potential within/beyond this research, and why? |

## Slide 2
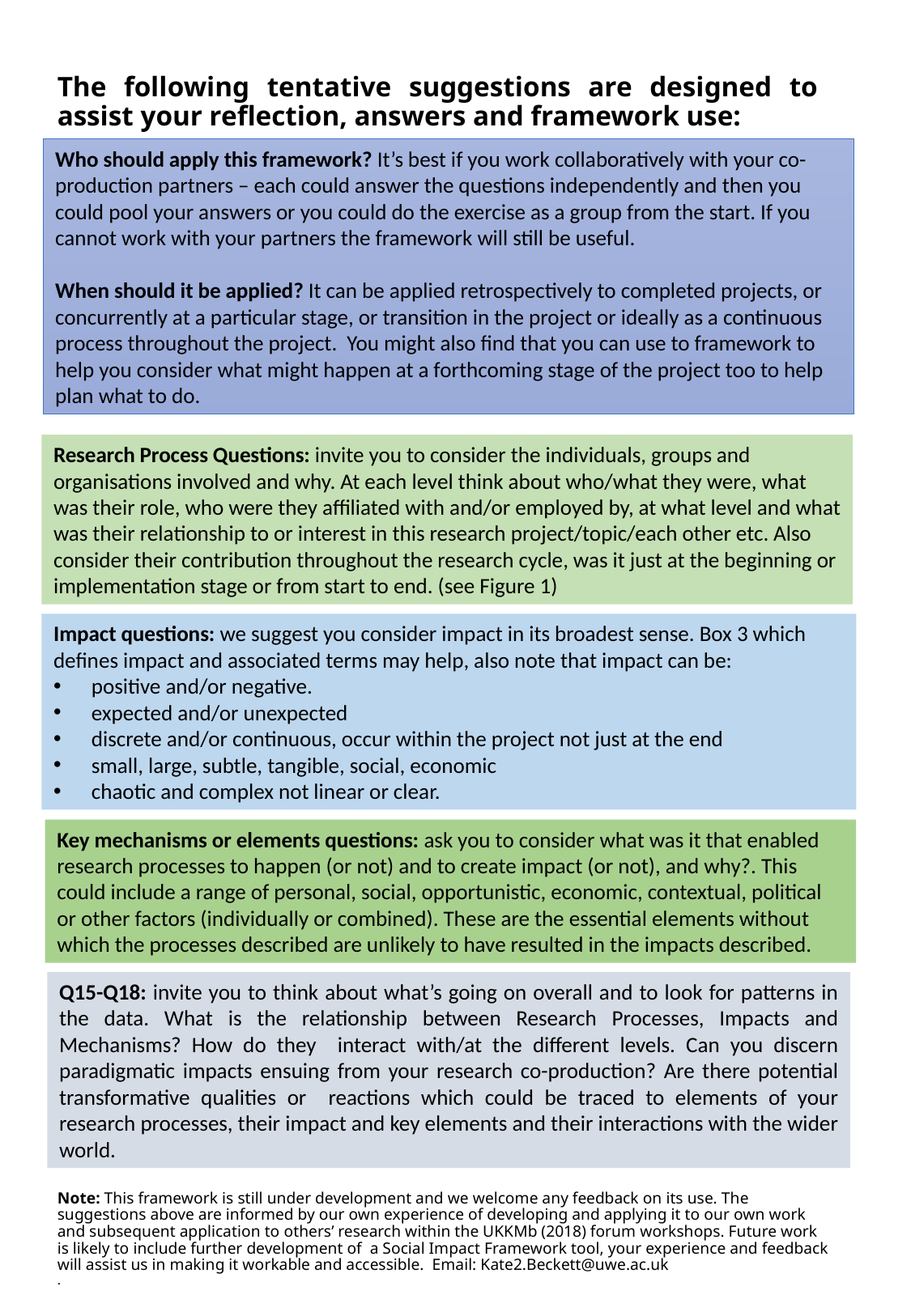

# The following tentative suggestions are designed to assist your reflection, answers and framework use:
Who should apply this framework? It’s best if you work collaboratively with your co-production partners – each could answer the questions independently and then you could pool your answers or you could do the exercise as a group from the start. If you cannot work with your partners the framework will still be useful.
When should it be applied? It can be applied retrospectively to completed projects, or concurrently at a particular stage, or transition in the project or ideally as a continuous process throughout the project. You might also find that you can use to framework to help you consider what might happen at a forthcoming stage of the project too to help plan what to do.
Research Process Questions: invite you to consider the individuals, groups and organisations involved and why. At each level think about who/what they were, what was their role, who were they affiliated with and/or employed by, at what level and what was their relationship to or interest in this research project/topic/each other etc. Also consider their contribution throughout the research cycle, was it just at the beginning or implementation stage or from start to end. (see Figure 1)
Impact questions: we suggest you consider impact in its broadest sense. Box 3 which defines impact and associated terms may help, also note that impact can be:
positive and/or negative.
expected and/or unexpected
discrete and/or continuous, occur within the project not just at the end
small, large, subtle, tangible, social, economic
chaotic and complex not linear or clear.
Key mechanisms or elements questions: ask you to consider what was it that enabled research processes to happen (or not) and to create impact (or not), and why?. This could include a range of personal, social, opportunistic, economic, contextual, political or other factors (individually or combined). These are the essential elements without which the processes described are unlikely to have resulted in the impacts described.
Q15-Q18: invite you to think about what’s going on overall and to look for patterns in the data. What is the relationship between Research Processes, Impacts and Mechanisms? How do they interact with/at the different levels. Can you discern paradigmatic impacts ensuing from your research co-production? Are there potential transformative qualities or reactions which could be traced to elements of your research processes, their impact and key elements and their interactions with the wider world.
Note: This framework is still under development and we welcome any feedback on its use. The suggestions above are informed by our own experience of developing and applying it to our own work and subsequent application to others’ research within the UKKMb (2018) forum workshops. Future work is likely to include further development of a Social Impact Framework tool, your experience and feedback will assist us in making it workable and accessible. Email: Kate2.Beckett@uwe.ac.uk
.
